# Sequential alteration of microglia and astrocytes in the rat thalamus following spinal nerve ligation

**DOI:** 10.1186/s12974-018-1378-z

**Published:** 2018-12-20

**Authors:** Lucie Blaszczyk, Marlène Maître, Thierry Lesté-Lasserre, Samantha Clark, Daniela Cota, Stéphane H. R. Oliet, Valérie S. Fénelon

**Affiliations:** 1 Bordeaux University, Bordeaux, France; 2 Neurocentre Magendie, INSERM U1215, Bordeaux, France

**Keywords:** Neuropathic pain, Ventral posterolateral thalamic nucleus, Intralaminar thalamic nuclei, Mediodorsal thalamic nucleus, GFAP, S100beta, Iba-1, Astrocytes, Microglia

## Abstract

**Background:**

Spinal reactive astrocytes and microglia are known to participate to the initiation and maintenance of neuropathic pain. However, whether reactive astrocytes and microglia in thalamic nuclei that process sensory-discriminative aspects of pain play a role in pain behavior remains poorly investigated. Therefore, the present study evaluated whether the presence of reactive glia (hypertrophy, increased number and upregulation of glial markers) in the ventral posterolateral thalamic nucleus (VPL) correlates with pain symptoms, 14 and 28 days after unilateral L5/L6 spinal nerve ligation (SNL) in rats.

**Methods:**

Mechanical allodynia and hyperalgesia (von Frey filament stimulation) as well as ambulatory pain (dynamic weight bearing apparatus) were assessed. Levels of nine glial transcripts were determined by quantitative real-time PCR on laser microdissected thalamic nuclei, and levels of proteins were assessed by Western blot. We also studied by immunohistofluorescence the expression of glial markers that label processes (GFAP for astrocytes and iba-1 for microglia) and cell body (S100β for astrocytes and iba-1 for microglia) and quantified the immunostained surface and the number of astrocytes and microglia (conventional counts and optical dissector method of stereological counting).

**Results:**

Differential, time-dependent responses were observed concerning microglia and astrocytes. Specifically, at day 14, iba-1 immunostained area and number of iba-1 immunopositive cells were decreased in the VPL of SNL as compared to naïve rats. By contrast, at day 28, GFAP-immunostained area was increased in the VPL of SNL as compared to naïve rats while number of GFAP/S100β immunopositive cells remained unchanged. Using quantitative real-time PCR of laser microdissected VPL, we found a sequential increase in mRNA expression of cathepsin S (day 14), fractalkine (day 28), and fractalkine receptor (day 14), three well-known markers of microglial reactivity. Using Western blot, we confirmed an increase in protein expression of fractalkine receptor at day 14.

**Conclusions:**

Our results demonstrate a sequential alteration of microglia and astrocytes in the thalamus of animals with lesioned peripheral nerves. Furthermore, our data report unprecedented concomitant molecular signs of microglial activation and morphological signs of microglial decline in the thalamus of these animals.

## Background

Neuropathic pain arises as a direct consequence of a lesion or disease affecting the somatosensory system [1]. Several mechanisms underlying neuropathic pain have been proposed. Among these, the implication of spinal glial cells in the peripheral neuropathic pain has been increasingly and consistently brought into focus. Spinal glial cells display changes in their morphology and their molecular repertoire following a peripheral nerve injury. Indeed, astrocytes become hypertrophied (more numerous and thicker processes as well as an enlarged cell body) with an increased expression of glial acidic fibrillary protein (GFAP) and S100β for instance [2–10] while microglial cells become hypertrophied and have increased ionized calcium binding adaptor molecule 1 (iba-1) expression [7, 11–13]. Under these morphological changes, astrocytes and microglia have been referred to as reactive or activated. In addition, astrocytes and microglia also release several inflammatory mediators [14, 15] that, in turn, increase spinal nociceptive neurons excitability while reinforcing glial activation, thereby contributing to neuronal sensitization and behavioral hypersensitivity observed in neuropathic pain. Activation of spinal microglia seems to precede activation of spinal astrocytes in peripheral neuropathic pain model [7, 16, 17]. Furthermore, microglia and astrocytes seem to play different roles in neuropathic pain. In fact, spinal microglia have been implicated in the initiation phase of peripheral nerve injury-induced pain [18–21]. Conversely, spinal astrocytes play a role in the maintenance of peripheral nerve injury-induced pain symptoms [10, 22].

The spinothalamic tract conveys information from spinal nociceptive neurons to higher order neurons in the contralateral ventral posterolateral thalamic nucleus (VPL), which is involved in sensory-discriminative aspects of pain processing [23], but also in the mediodorsal thalamic nucleus (MD) and the group of intralaminar thalamic nuclei (IM), which are important in processing affective components of pain. It has been shown that chronic constriction injury (CCI) of the sciatic nerve in rats induces 10 days later an alteration in the Nav1.3 sodium channel mRNA expression within VPL neurons contributing to increased excitability of VPL neurons and to neuropathic pain [24]. In the same pain model, microglial activation was found in the VPL and injection into the VPL of a microglial inhibitor was able to transiently abrogate pain symptoms [25]. However, although VPL neurons sensitize to mechanical stimuli in a well-established peripheral neuropathic pain model in rodents, the spinal nerve ligation (SNL) model [26], nothing is known about a possible involvement of thalamic glial cells in this sensitization. Therefore, we designed this study in order to explore a possible activation of astrocytes and microglia in thalamic nuclei involved in pain processing using the SNL model. Mechanical allodynia and hyperalgesia as well as ambulatory pain were assessed. We explored mRNA levels of up to nine genes of structural and functional glial markers in microdissected thalamic nuclei by using quantitative real-time PCR. We tested whether changes in mRNA were also found at the protein level by using Western blot. We also studied by immunohistofluorescence the expression of some astrocytic and microglial markers so to reveal potential glial activation. We aimed at determining whether glial cells were hypertrophied and whether the number of glial cells was altered. Therefore, we used glial markers that label processes (GFAP for astrocytes and iba-1 for microglia) and cell body (S100β for astrocytes and iba-1 for microglia). These studies were done at two time points after the surgery (day 14 and day 28), to evaluate a possible sequential activation of microglia and astrocytes, as already observed at the spinal cord level.

## Materials and methods

### Animals and study design

All experiments followed the ethical guidelines of the International Association for the Study of Pain and the European Community Council directive of 22 September 2010 (2010/63/EU). The project was approved by Bordeaux Ethical Committee (CEEA 50) under n° 5012039-A. All efforts were made to minimize the number of animals used. Adult male Wistar rats (150–175 g) obtained from Janvier (France) were used in this study. Animals were housed two per cage in a controlled environment under a 12-h light/dark cycle (lights on 07:00–19:00, 22 °C) with unrestricted access to water and food. Rats were acclimatized for 4 days to the animal facility and for 3 days to manipulations and devices prior to behavioral studies.

Three groups of animals were used: rats with surgery and ligation of L5 and L6 spinal nerves (SNL, *n* = 47), rats with surgery but no ligation (sham, *n* = 44), and rats with no treatment (naïve, *n* = 34). Seven to eight rats in each group (*n* = 7 in sham and *n* = 8 in naïve and SNL) were used to quantify mRNA on laser microdissected thalamic nuclei at day 14 and eight to nine rats in each group (n = 8 in sham and *n* = 9 in naïve and SNL) at day 28. Four naïve, six sham, and six SNL were used to study microglia with immunohistochemical techniques at day 14 while five naïve, four sham, and five SNL were used at day 28. Four naïve, five sham, and six SNL were used to study astrocytes with immunohistochemical approaches at day 14 as well as at day 28 as well as to assess changes in protein expression by using Western blot on formalin-fixed tissues. Behavioral tests were performed on all these animals to the exception of four naive. In addition, eight sham and eight SNL at day 14 were only subjected to behavioral tests. The first time point was set at 14 days after the surgery since pain symptoms and sensitization of VPL neurons are well established at this time in the SNL model [26]. The second time point was set at day 28 after the surgery since a previous study reported a remote microglial activation within the VPL 16 and 28 days after sciatic nerve injury [27].

### Surgery

The well-established L5–L6 spinal nerve ligation rodent model of neuropathy was used in this study [28]. Rats were deeply anesthetized under gaseous anesthesia with a 1:1 flow ratio of air/O_2_ (1 L/min) containing 5% isoflurane (IsoFlo®, Axience, Pantin, France). Once the anesthesia was induced, the maintenance was performed with a 1:1 flow ratio of air/O_2_ (0.5 L/min) containing 2% isoflurane. A local anesthesia was also performed with lidocaine (Lurocaïne®, 20 mg/mL, Vétoquinol N.-A Incorporation, Lavaltrie, Canada) before skin incision and at the time of wound closing. Briefly, the skin of the back was incised longitudinally, the transverse processes of the sixth lumbar vertebra was excised, and the right L5 and L6 spinal nerves were isolated and tightly ligated with a 6.0 polyamid thread (Ethicon, Issy les Moulineaux, France). After complete hemostasis, the incision was closed first by ligating muscular plane with a 4.0 nylon thread (Ethicon) then by using six to seven metal skin clips (AMS, Bordeaux, France). Then, lidocaine was subcutaneously injected in the operated region, and 100 μL of antibiotics was injected intramuscularly in the left thigh (7.5% of Borgal®, Virbac, Carros, France). Rats were housed in an individual cage for 48 h and followed up to quantify their general state and detect the possible presence of surgical complications or abnormal excessive pain. Metal skin clips were removed at day 7 post-surgery under a brief gaseous anesthesia, following the same procedure as before. Sham rats underwent exactly the same procedure, but the exposed spinal nerves were not ligated. Any structure located on the right operated side will be called “ipsilateral” and any structure located on the opposite side, “contralateral.”

### Behavioral tests

All behavioral tests were done by the same female experimenter and within the same time windows at day 0 (D0) prior to surgical procedure, at day 14 (D14) after surgery (or at age-matched time points for naïve animals), and at day 28 (D28) before sacrifice.

Mechanical allodynia and hyperalgesia were measured at both hind paws of the rats using responses to von Frey filaments (0.008–100 g) stimulation according to the method previously described in Ducourneau et al. [5]. Each von Frey filament was applied on the plantar surface of the hind paw five times with at least a 5-s interval and in ascending order. The rat response was scored by an experimenter blind to the experimental group (except for the naïve group which had no scar) according to the following elements: (score 1) detection, rats slightly contracts their leg muscles without any actual movement; (score 2) withdrawal reaction; (score 3) escape, rats withdraw and avoid further contact with the stimulus, by moving their body away from the stimulus; and (score 4) licking/biting of the stimulated paw after withdrawal. Score was equal to zero in case of absence of response. A mean score value was then calculated for each von Frey Filament. Since each von Frey filament was applied five times, the first mean score value above detection was 1.2 and was used to calculate the nociceptive response-threshold by a simple linear approximation. Accordingly, this score value corresponds to the stimulus intensity at which more than 50% of withdrawal responses could be observed over five responses. To quantify mechanical hyperalgesia, we used a hyperalgesia score calculated as the difference between the areas under the response curve obtained at D14 (or D28) and at D0. In both cases, areas were measured from above the value of the threshold that was measured at day 0 and from above 1.2 behavioral score value. This score was mathematically quantified using an Excel macro for both ipsilateral and contralateral hind paw of each tested animal. When a negative score was obtained, it was set at zero.

Ambulatory pain was evaluated by using the dynamic weight bearing (DWB, Bioseb, France) apparatus as extensively described in Ducourneau et al. [5]. Briefly, a mean value of the weight bore by each limb all over the experiment was calculated for the whole validated testing period and expressed as percentage of the animal weight.

### Brain tissue samples

For quantitative real-time PCR approach, rats were deeply anesthetized under gaseous anesthesia with a mixture of isoflurane (5%) and a 1:1 flow ratio of air/O_2_ (1 L/min) and then decapitated. The brain was rapidly frozen on dry ice and stored at − 80 °C until sectioning was done. For sectioning, the frozen brain was mounted onto Tisue-Tek® OCT compound on a chuck and the brain was placed in a Leica CM3050S cryostat (Leica Microsystems, Wetzlar, Germany) for 20 min to equilibrate the brain with a chamber temperature of − 20 °C and an object temperature of − 18 °C. Sixty-micrometer-thick sections of the thalamus region were cut and placed on polyethylene naphthalate membrane slides (Carl Zeiss, Munich, Germany) under RNAse-free conditions. The slides were immediately transferred into ice-cold 95% ethanol (Merck, USA) for 40 s and incubated in 75% ethanol for 30 s and in 50% ethanol for 30 s. Specimens were briefly stained in 1% cresyl violet solution. Tissue sections were dehydrated through 50% ethanol (30 s), 75% ethanol (30 s), and 95% ethanol (30 s), followed by two 40-s incubation in anhydrous 100% ethanol. Slides were dried for 5 min at room temperature. Immediately after dehydration, laser-assisted microdissections were performed using a P.A.L.M. MicroBeam microdissection system version 4.8 equipped with a P.A.L.M. RoboSoftware (P.A.L.M. Microlaser Technologies AG, Bernried, Germany). Microdissection was performed at × 5 magnification. Samples of VPL on both sides and of the ventral posteromedian thalamic nucleus (VPM) on the contralateral side were collected in adhesives caps (P.A.L.M. Microlaser Technologies AG, Bernried, Germany). Both VPL were collected as some bilateral projections have been reported in a model of unilateral hemi section of the thoracic spinal cord [29]. The contralateral VPM was chosen as a control thalamic nucleus as it receives only nociceptive information from the head and skull. To limit RNA degradation, samples were collected for up to 30 min per slide, after which the caps were placed on a sterile microcentrifuge tube containing 350 μl of lysis buffer. The samples were then stored at − 80 °C until extraction was done.

For Western blot, histochemistry, and immunohistochemical detection of glial markers, rats were killed under deep anesthesia with pentobarbital (100 mg/kg i.p.) and perfused transcardially with warm (37 °C) heparinized saline (25 IU heparin/mL; 2–3 min) followed by cold (10 °C) phosphate-buffered solution (0.1 M, pH 7.6) containing 4% (for astrocytic markers) or 2% (for microglial marker) paraformaldehyde and 0.4% picric acid for 30 min. Brains were then removed and transferred into 0.05 M Tris buffered saline (TBS) containing 30% sucrose for cryoprotection and 0.05% sodium azide for conservation and left at 4 °C until use. Coronal sections (30-μm-thick) were cut on a cryomicrotome (Microm, Germany). Slices were then stored in culture plate wells within the TBS/30% sucrose/0.05% sodium azide solution at 4 °C until use. Slices were sequentially deposited in eight wells; therefore, in each well, slices were 240 μm apart. Each sequence of cutting included animals from different groups to homogenize experimental conditions. The ipsilateral side of the brain cortex was gently notch to determine the side of the brain for the future quantifications.

### Quantitative real-time PCR

Total RNA was extracted from microdissected tissues using the RNeasy® micro Kit (Qiagen, Hilden, Germany) according to the manufacturer’s protocol and eluted with 14 μl of RNAse-free water. All samples displayed RIN greater than or equal to 7.8 as determined using the RNA 6000 Pico Kit and the bioanalyzer 2100 (Agilent Technologies). cDNA was synthesized from 90 ng of total RNA using RevertAid Premium Reverse Transcriptase (Fermentas) and primed with oligo-dT primers (Fermentas) and random primers (Fermentas). PCR amplification was performed using a LightCycler® 480 Real-Time PCR System (Roche, Meylan, France) with following cycles (95 °C for 5 min followed by 45 cycles with 95 °C, 15 s and 61 °C for 30s). Quantitative real-time PCR (Q-PCR) reactions were done in duplicate for each sample, using transcript-specific primers, cDNA (1 ng), and LightCycler 480 SYBR Green I Master (Roche) in a final volume of 10 μl. Due to the small amount of collected tissue, we were able to explore mRNA expression of only nine genes. Primer sequences used are reported in Table 1. We choose to study the mRNA expression of three genes that code for proteins that are usually used as astrocytic markers in immunohistofluorescence: GFAP and S100β protein that are enriched in astrocytes [30] and upregulated in reactive astrocytes [31] as well as the glutamine synthetase that is an astrocytic enzyme that generates glutamine from glutamate and ammonia. To the best of our knowledge, only a short communication has reported in 1990 a non-astrocytic glutamine synthetase expression in the rat brain based only on shape, size, and location of nucleus of the glutamine synthetase expressing cells [32]. Therefore, as in humans [33], it appears reasonable to consider the glutamine synthetase as an astrocytic-specific marker in the rat brain, which is not the case in the mouse brain [34, 35]. For the microglial markers, we studied the mRNA expression of the genes coding for three proteins that are specifically expressed in microglia and the expression of which has been shown to be altered in reactive microglia: iba-1 [36, 37], the cluster of differentiation molecule 11 b (CD11b) [38, 39], and the fractalkine receptor (CX3CR1) [40–43]. As CX3CR1 is involved in a well-defined microglia-neuron-microglia interaction loop, we added the genes of the other protagonists. Indeed, the fractalkine (a chemokine named CX3CL1 and reported as “other” among the astrocytic and microglial markers in the figures) is expressed on neuronal membrane and cleaved by cathepsin S (CTSS), a cysteine protease expressed and secreted by microglia. The obtained soluble form of CX3CL1 acts on the fractalkine receptor expressed on microglia. Finally, we added the toll-like receptor 4 (TLR4) as it is a specific receptor for lipopolysaccharide known as a signal for microglia activation and as microglia is the only central nervous system glial cells that express TLR4 [44]. PCR data were exported and analyzed in an informatics tool (Gene Expression Analysis Software Environment) developed at the NeuroCentre Magendie. For the determination of the reference gene, the Genorm method was used [45]. Relative expression analysis was corrected for PCR efficiency and normalized against two reference genes: peptidypropyl isomerase A and succinate dehydrogenase complex subunit A genes for microdissected thalamic nuclei at day 14 and valosin-containing protein and β actin genes for microdissected thalamic nuclei at day 28. The relative level of expression was calculated using the comparative (2^−∆∆CT^) method [46], and naïve animals were arbitrarily set at 1. The results are presented as color-coded tables (for example see Fig. 3) showing the ΔCt obtained for each of the tested genes (column, ordered in categories) and for each animal (row, ordered in the three experimental groups: naïve, sham, SNL). The smaller the ΔCt (yellow), the higher the mRNA expression is; the higher the ΔCt (deep blue), the smaller the mRNA expression is. The median value of the color code (light blue) corresponds to the mean of the ΔCt obtained in naïve animals for a given gene. It must be noted that, on average, the difference between high (deep blue) and low value (yellow) is 2.35. When no result was obtained, the cell appears white. When the treatment has a significant effect on the mRNA expression of a given gene, histogram showing the fold change is presented.

**Table 1:** Quantitative real time PCR primers

Gene name	Primer name	GenBank ID	Forward primer sequence	Reverse primer sequence
Glial fibrillary acidic protein	Gfap	NM_017009	GGTGTGGAGTGCCTTCGTATTAG	GGGACACTTTCAGCTCCATTTCT
S100 calcium binding protein, beta polypeptide	S100b	NM_013191	GAGGAATGAAGGGCCACTGA	CCCTAGGCACCAGCAGGTC
Glutamine synthetase	GS	NM_017073	ATTCCACGAAACCTCCAACATC	TCTCCTGGCCGACAATCC
Cluster of differentiation 11b/integrin alpha M	CD11b/Itgam	NM_012711	TGAAGGCTCAGACAGAGACCAAA	GCTGCCCACAATGAGTGGTAC
Ionized binding adaptor molecule 1	Iba-1	NM_017196	CAACAAGCACTTCCTCGATGATC	TGAAGGCCTCCAGTTTGGACT
Toll-like receptor 4	Tlr4	NM_019178	AGCAATGTGATGAAACCCCATAC	GATTCTTTGCCTGAGTTGCTTAATT
Cathepsin S	Ctss	NM_017320	GTGGTTGGCTATGGGACTCTTG	CCAAAGTGAAGGCCCCAACT
Chemokine (C-X3-C motif) ligand 1/fractalkine	Cx3cl1	NM_134455	CAGCCAGTGACTCACTCCTTGA	CTGGCTTCCTCACTCTGGGA
Chemokine (C-X3-C motif) receptor 1/fractalkine receptor	Cx3cr1	NM_133534	GAACCGGAAGAAGGCCAGAG	GCGTCCAGAAGAGGAAGAAGAC
β actin	Actb	NM_031144	CGGCAATGAGCGGTTCC	TGCCACAGGATTCCATACCC
Peptidylprolylisomerase A	Ppia	NM_017101	ACCCCATCTGCTCGCAATAC	GGAATGAGGAAAATATGGAACCC
Succinate dehydrogenase complex, subunit A	Sdha	NM_130428	TGCGGAAGCACGGAAGGAGT	CTTCTGCTGGCCCTCGATGG
Valosin-containing protein	Vcp	NM_053864	GAGGTTTTGGCAGCTTCAGATT	ACCTCCACTGCCCTGACTTG

### Western blot

We used 20 free-floating sections (30-μm-thick) per animal to collect tissue samples. We mounted each section on a glass slide, and by using a disposable biopsy punch (Kai medical, 2 mm diameter, Seki, Japan), we collected a piece of the contralateral thalamus including the VPL. Therefore, we obtained around 1.88 mm^3^ formalin-fixed tissue/per animal.

Protein was extracted using the Qproteome FFPE Tissue Kit (Qiagen), and then quantified using the Lowry method (Biorad RC DC Protein Assay Kit, France) following the manufacturer’s instructions.

Thirty microgram of protein was loaded onto 4–15% precast polyacrylamide gels and transferred to PVDF membranes (Biorad) using the Transblot Turbo System (Biorad). The resulting blot was blocked for 1 h at room temperature with 5% fat-free milk in Tris-buffered saline containing Tween 20 then incubated overnight at 4 °C with the following primary antibodies: rabbit anti-CX3CR1 (1/1000; catalog n° TP501; Torrey Pines Biolabs, Secaucus, USA; RRID: AB_10892355) or rabbit anti-beta actin (1/1000; catalog n° 4967; Cell Signaling, France; AB_330288) diluted in 5% bovine serum albumin in Tris-buffered saline containing Tween 20.

After washing, membranes were incubated with a horseradish peroxidase-conjugated secondary antibody (goat anti-rabbit 1/2000; catalog n°7074; Cell Signaling; AB_2099233). Immunoreactive bands were visualized using enhanced chemiluminescence (Western Lightning ECL plus, PerkinElmer, France) and detected using the Biorad Chemidoc Touch imaging system.

Between antibodies, membranes were stripped with a solution containing β-mercaptoethanol and re-blocked with fat-free milk. Quantitative analysis was performed by densitometry with ImageJ software (NIH, Bethesda, MA) and normalized with the loading control beta-actin.

### Histochemistry

We used a histochemical method for localizing acetylcholinesterase activity via acetylthiocholine in order to properly delineate thalamic nuclei (see Figs. 6a and 7a).

Sections (from an adjacent well of the one treated for the immunohistofluorescent detection of glial markers) were incubated in sodium acetate buffer (0.2 M, pH 5.9) for 5 min and then for 15 min in a pre-incubation solution under gentle agitation (100 ml 0.2 M sodium acetate buffer at pH 5.9, 0.25 g copper sulfate pentahydrate and 0.375 g glycine). Acetylcholinesterase incubating medium (100 mg acetylthiocholine iodide in the pre-incubation solution) was prepared immediately before use, and slides were incubated for 4 h at room temperature under gentle agitation and then washed in sodium acetate buffer for 5 min. To visualize the sites of acetylcholinesterase activity, the sections were subjected to a 1% ammonium sulfate solution (in sodium acetate buffer) for 2 min. The sections were then rinsed three times in sodium acetate buffer for 5 min, mounted on gelatin-coated slides, coverslipped using Vectashield (Vector Laboratories, Inc., Burlingame, CA), and then stored at 4 °C until use.

### Immunohistochemistry

All immunofluorescence steps were performed under gentle agitation at room temperature. Slices were rinsed three times in 0.05 M TBS for 10 min and then incubated with primary antiserum overnight. After incubation, slices were washed again three times in TBS for 10 min and incubated in secondary antibody solution for 2 h in obscurity. Primary antibodies were diluted in TBS containing 0.25% Triton X-100 and normal goat serum (10% for immunohistochemical detection of astrocytes or 1% for immunohistochemical detection of microglia). Secondary antibodies were diluted in TBS containing 0.25% Triton X-100 and the nuclear marker 4′,6′-diamidino-2-phenylindol (DAPI; 1:10,000; catalog n° D1306, Molecular Probes, Life Technologies, Carlsbad, California, USA). Slices were mounted on gelatin-coated slides and coverslipped using Vectashield (Vector Laboratories, Inc., Burlingame, CA), then stored at 4 °C until use. All animals and all groups were run simultaneously: one well per animal, all animals per condition.

For visualization of microglia, we used a single immunohistofluorescent technique. Sections were incubated in a rabbit antiserum raised against a synthetic peptide corresponding to the C-terminus of iba-1 (1:2000; catalog n° 019-19741; Wako, Osaka, Japan; AB_839504) and then in a goat anti-rabbit secondary antibody conjugated to Alexa 488 (1:2000; Jackson Immunoresearch Laboratories, West Grove, USA).

For visualization of astrocytes, we used a double immunohistofluorescent technique. We incubated sections in a rabbit antiserum against GFAP (1:1000; catalog n° Z0334; AB_10013382; Dako, Trappes, France) and a monoclonal mouse antibody against protein S100β-subunit (S100β; 1:10,000; clone SH-B1; catalog n° S2532; AB_477499; Sigma-Aldrich, St-Louis, MO, USA). Then, species-specific secondary antibodies which have been raised in goat (anti-rabbit conjugated to cyanine 3 diluted 1:300 and anti-mouse conjugated to Alexa 488 diluted 1:2000) were used and were supplied by Jackson Immunoresearch Laboratories (West Grove, USA).

### Imaging and analysis of stained sections

Quickly after histochemistry or the immunofluorescence treatment, stained sections were examined with a Leica DMI6000 spinning disk microscope (Leica Microsystems, Wetzlar, Germany) and digital images were captured with a HQ2 CCD camera (Photometrics, Tucson, USA) under a × 20 objective (HC PL APO CS, 0.7 NA, Leica Microsystems, Wetzlar, Germany). The diode lasers used were at 408 nm (for DAPI), 491 nm (for Alexa 488), and 561 nm (for cyanine 3). The technique of scan slide was used in order to visualize and reconstruct all areas of interest. The mosaics were done with a motorized stage Scan IM (Märzhäuser, Wetzlar, Germany). This system was controlled by MetaMorph software (Molecular Devices, Sunnyvale, USA). Parameters were determined at the beginning of the acquisition and were maintained for all acquisitions of the same experiment. For each animal, three non-adjacent slices were randomly selected and acquired. For each slice, two images (ipsilateral and contralateral side) of the lateral thalamus containing the VPL and the VPM were captured as well as an image of the central thalamus containing the MD and the IL.

For GFAP and iba-1, morphometric analysis was performed with the Metamorph 7.8.1.0 program. The procedure consisted of three steps: (1) background substraction, (2) thresholding, and (3) morphometric calculations in delineated areas (ipsi- and contralateral VPL as well as its subregion, contralateral VPM, MD, and IL). As the VPL presents a fine topographic map of the fore- and hindlimbs [47], we delineate the subregion of the VPL (see Fig. 6A) that receives information from the hindlimb (we call it VPL subregion). Thresholding of images was made to obtain positive versus negative pixel in a 0-65535 gray scale. Delineation of regions of interest was performed using stereotaxic coordinates [48] and cytoarchitectonic boundaries gathered on sections treated to reveal the activity of the acetylcholine esterase (see Figs. 6a and 7a). For each section, the total surface of each delineated area as well as the total immunostained surface within each delineated area was calculated. Values of immunostained surface were expressed in percent, and for each rat, the mean of percent values obtained on three different slices was calculated for each area of interest.

For astrocytes and microglia conventional counts, manual count of GFAP/S100β/DAPI or iba-1/DAPI-positive cells in ipsi- and contralateral VPL subregion was performed with the Metamorph 7.8.1.0 program following the previously described determination of immunostained surface. Microglia were identified based on having ramified iba-1-labeled fibers (green) emanating from a iba-1-stained cell body (green) displaying a DAPI-stained nucleus (deep blue resulting in a turquoise part of the cell body; see Fig. 6B). Astrocytes were identified based on having at least two thick GFAP-labeled fibers (red) emanating from a S100β-stained cell body (green) displaying a DAPI-stained nucleus (deep blue resulting in a turquoise part of the cell body; see Fig. 9a). Therefore, for each rat, we calculated the mean of the GFAP/S100β/DAPI-positive or iba-1/DAPI-positive cell number per surface area unit (μm^2^) obtained in the three different sections.

In order to verify that our conventional method of counting did not introduce any bias, we performed an optical disector method of stereological counting on Nanozoomer acquired iba-1 immunostained sections. The slide scanner was a Nanozoomer 2.0HT with fluorescence imaging module (Hamamatsu Photonics France) using objective UPS APO 20× NA 0.75 combined to an additional lens × 1.75, leading to a final magnification of × 35. Virtual slides were acquired with a TDI-3CCD camera. Fluorescent acquisitions were done with a mercury lamp (LX2000 200 W - Hamamatsu Photonics, Massy, France) and the set of filters adapted for Alexa 488 fluorescence. Iba-1 immunopositive cell density was determined using the optical disector with the stereological module of Mercator (Explora Nova, La Rochelle, France) on six images of sections that were at least 210 μm apart, for each animal. Within the delineated VPL, the computer program automatically generates counting frames, which measured 50 μm × 50 μm (width × height) and are spaced in *X*- and *Y*-axis by 30 μm. The disector frame consists of two in-bound green lines (inclusion lines) and two out-of-bounds red lines (exclusion lines). To the exclusion of the cells that touched the red lines, the investigator counted cells within each counting frame. The total number of counted iba-1 immunopositive cells was then divided by the total volume of the counting frames to determine iba-1 immunopositive cell density (number/mm^3^).

All analyses were performed by an experimenter who was blinded to the experimental group. To the exception of the optical disector method of stereological counting, all analyses were performed on a single focal plane.

For preparing illustrations, selected images were processed using Adobe Photoshop CS (Adobe Systems, Mountain View, CA). Only brightness and contrast were adjusted for the whole frame, and no part of a frame was enhanced or modified in any way.

### Statistical analysis

All data were analyzed using a scientific graphing and data analysis software GraphPad Prism 5 (GraphPad Software). When the KS normality test failed, a Kruskal-Wallis one-way analysis of variance (ANOVA) on ranks was performed followed by an all pairwise multiple comparison procedures (Dunn’s method). When the KS normality test passed, one-way ANOVA followed by Tukey’s post hoc test was used. Linear relationships were assessed using Pearson’s correlation test. A *p* level of 0.05 was set as the level of statistical significance. As naïve animals were arbitrarily set at 1 in the figures of PCR results, all other data (behavior and immunohistochemistry) were also presented as percentage of naïve. All data are expressed as mean ± SEM.

## Results

### Pain behavior following spinal nerve ligation

**Fig. 1:**
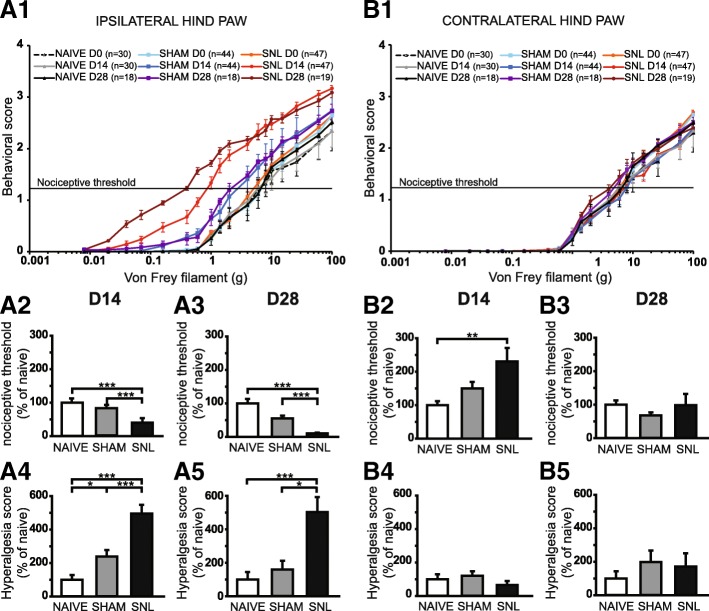
Mechanical static allodynia and hyperalgesia impairment 14 and 28 days after spinal nerve ligation. The mean behavioral score in response to five stimuli applied on the plantar surface of the ipsilateral (**A1**) and contralateral (**B1**) hind paw of naïve, sham, and spinal nerve-ligated (SNL) animals at day 0 (D0), day 14 (D14), and day 28 (D28) is plotted against the strength of the applied von Frey hair. Note that the curves of SNL at D14 and D28 for the ipsilateral hind paw are dramatically shifted to the left and to the top compared to D0 (**A1**). Bar histograms show mean nociceptive threshold (expressed as percentage of naïve values) of the ipsilateral hind paw at D14 (**A2**) and D28 (**A3**) and of the contralateral hind paw at D14 (**B2**) and D28 (**B3**) as well as the hyperalgesia score of the ipsilateral hind paw at D14 (**A4**) and D28 (**A5**) and of the contralateral hind paw at D14 (**B4**) and D28 (**B5**). The nociceptive threshold of the ipsilateral hind paw of the SNL rats at D14 and D28 was significantly reduced compared to the ones of the naïve and of the sham rats (**A2**: Kruskal-Wallis test *H*(2) = 44.45, *p* < 0.0001; **A3**: Kruskal-Wallis test *H*(2) = 33.79, *p* < 0.0001). For the contralateral hind paw, the nociceptive threshold of SNL rats was significantly increased at D14 compared to the one of the naïve rats (**B2**: Kruskal-Wallis test *H*(2) = 11.87, *p* = 0.0026) but not at D28 (**B3**: Kruskal-Wallis test *H*(2) = 3.95, *p* = 0.14). The hyperalgesia score (expressed as percentage of naïve values) of the ipsilateral hind paw of the SNL rats at D14 and D28 was significantly increased compared to the ones of the naïve and of the sham rats (**A4**: Kruskal-Wallis test *H*(2) = 37.34, *p* < 0.0001; **A5**: Kruskal-Wallis test *H*(2) = 18.30, *p* = 0.0001). Note that the hyperalgesia score of the ipsilateral hind paw of the sham rats is significantly higher than the one of the naïve rats at D14 (**A4**). No hyperalgesia was detected on the contralateral hind paw (**B4**: Kruskal-Wallis test *H*(2) = 5.36, *p* = 0.0683; **B5**: Kruskal-Wallis test *H*(2) = 1.328, *p* = 0.51). Post hoc test (Dunn’s multiple comparison test): *p < 0.05; **p < 0.01; ***p < 0.001

Overall, SNL rats display a progressive (D14 and then D28) and dramatic reduction in nociceptive threshold to von Frey hair stimulation on the ipsilateral hind paw (Fig. 1A1). Indeed, SNL curves displayed a shift to the left between D0 and D14 and also between D0 and D28: previously non painful stimuli became painful, a process known as allodynia. Consistently, the nociceptive threshold of the ipsilateral hind paw of SNL rats was significantly reduced at D14 (Fig. 1A2) and also at D28 (Fig. 1A3) compared to that of the naïve (60 and 90% decrease respectively) and of the sham rats (51% and 80% decrease respectively). The decrease of nociceptive threshold was paralleled by an increase in the intensity of the behavioral responses to von Frey hair stimulation above and below the initial nociceptive threshold (Fig. 1A1). Indeed, SNL curves displayed a shift to the top between D0 and D14 and also between D0 and D28: a previously painful stimulus produced a more painful reaction, a process known as hyperalgesia. Accordingly, the hyperalgesia score of the ipsilateral hind paw of SNL rats was dramatically and significantly increased at D14 (Fig. 1A4) but also at D28 (Fig. 1A5) compared to that of the naïve (5 times greater at both time points) and of the sham rats (2 and 3 times greater respectively). It is of interest to note that the hyperalgesia score of the ipsilateral hind paw of sham rats is significantly higher compared to that of the naïve rats at D14 (2.4 times greater; Fig. 1A4).

For the contralateral hind paw, no obvious shifts to the left or to the top of the SNL curves were observed (Fig. 1B1). Accordingly, the analysis of the variance of the nociceptive threshold at D28 (Fig. 1B3) as well as of the hyperalgesia score at D14 (Fig. 1B4) and D28 (Fig. 1B5) revealed no effect of the treatment. Surprisingly, the nociceptive threshold of the contralateral hind paw of SNL rats at D14 was significantly higher compared to that of the naïve rats (2.3 times greater; Fig. 1B2). Therefore, a previously painful stimulus at D0 could become non-painful at D14.

**Fig. 2:**
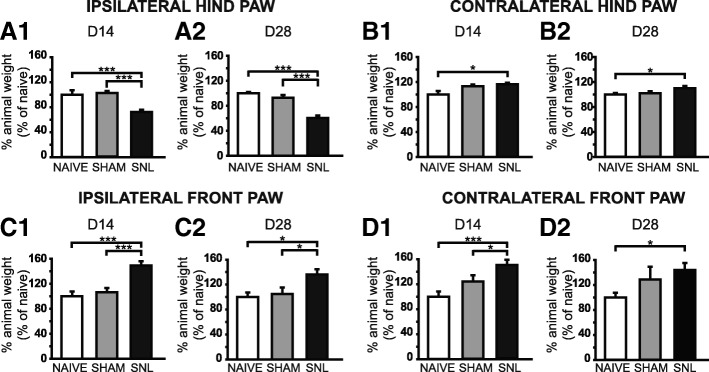
Weight-bearing impairment 14 and 28 days after spinal nerve ligation. Bar histograms show the percentage of animal weight (expressed as percentage of naïve values) distributed on the ipsilateral (**A1** and **A2**) and contralateral (**B1** and **B2**) hind paws as well as the ipsilateral (**C1** and **C2**) and contralateral (**D1** and **D2**) front paws in naïve, sham, and SNL rats at D14 (**A1**, **B1**, **C1** and **D1**) and D28 (**A2**, **B2**, **C2** and **D2**). The percentage of the animal weight borne on the ipsilateral hind paw in SNL animals was significantly decreased at D14 (**A1**: Kruskal-Wallis test *H*(2) = 35.73, *p* < 0.0001) and D28 (**A2**: Kruskal-Wallis test *H*(2) = 28.87, *p* < 0.0001) compared to sham and naïve rats while the percentage of the animal weight borne on the ipsilateral front paw was significantly increased at D14 (**C1**: Kruskal-Wallis test *H*(2) = 27.89, *p* < 0.0001) and D28 (**C2**: Kruskal-Wallis test *H*(2) = 9.832, *p* = 0.0073) compared to sham and naïve rats. The percentage of the animal weight borne on the contralateral hind paw in SNL animals was significantly increased at D14 and D28 (**B1**: Kruskal-Wallis test *H*(2) = 7.449, *p* = 0.0241; **B2**: Kruskal-Wallis test *H*(2) = 8.024, *p* = 0.0181) compared to naïve animals. This was also true for the contralateral front paw (**D1**: Kruskal-Wallis test *H*(2) = 14.39, *p* = 0.0007; **D2**: Kruskal-Wallis test *H*(2) = 6.682, *p* = 0.03). In addition, the percentage of the animal weight borne on the contralateral hind paw in SNL animals was also significantly increased at D14 compared to naïve animals. Post hoc test (Dunn’s multiple comparison test): *p < 0.05; ***p < 0.001

DWB evaluation showed a reduced use of the ipsilateral hind paw in SNL rats at D14 (Fig. 2A1) and D28 (Fig. 2A2) with a significant drop of the weight borne compared to that of naïve (28% and 41% decrease respectively) and sham rats (28% and 37% decrease). The load was redistributed to the contralateral hind paw (Fig. 2B1, B2) as well as the front paws (Fig. 2C1, C2, D1, and D2) with a significant increase of the weight borne compared to that of the naïve rats at D14 and D28 (1.2 times greater for the contralateral hind paw and around 1.5 times greater for the front paws). The difference between the weight borne in sham and SNL rats only reached statistical significance for the ipsilateral front paw at D14 (1.5 times greater; Fig. 2C1) and D28 (1.2 times greater; Fig. 2C2) and for the contralateral front paw at D14 (1.2 times greater; Fig. 2D1).

In conclusion, SNL animals display mechanical static allodynia and hyperalgesia as well as a reduced use of the ipsilateral hind paw 14 days as well as 28 days after the L5–L6 spinal nerve ligation. The surgery by itself (without the nerve ligation) produces a transient mechanical static hyperalgesia 14 days after the surgery.

### Alteration in thalamic mRNA expression 14 and 28 days after spinal nerve ligation

**Fig. 3:**
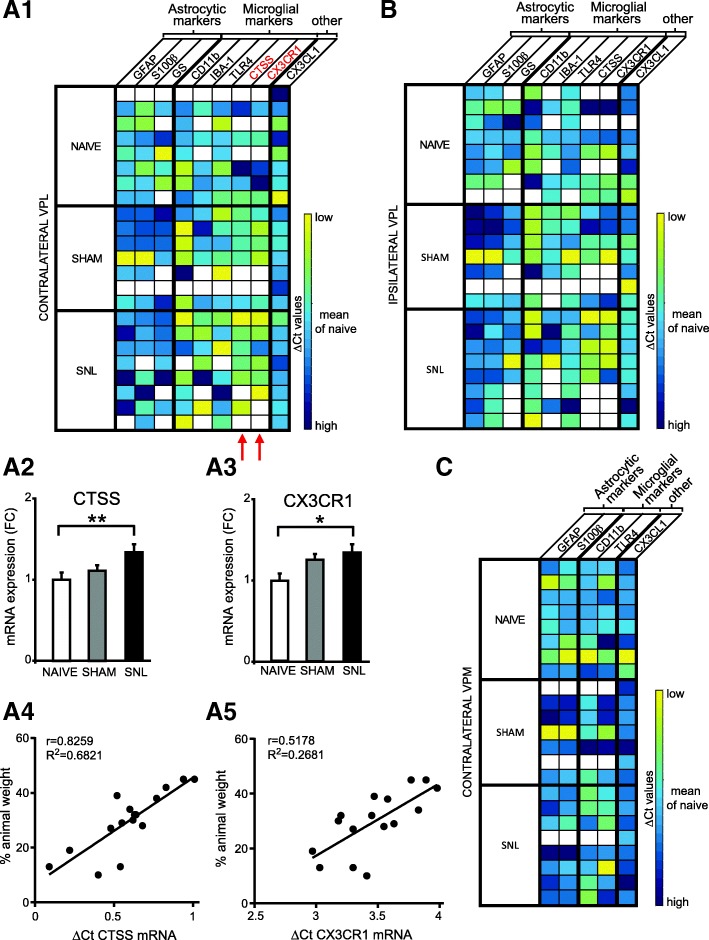
Upregulation of microglial markers’ mRNA expression in microdissected contralateral VPL 14 days after spinal nerve ligation. Tables summarize the expression of up to nine genes on microdissected contralateral (**A1**) and ipsilateral (**B**) VPL as well as contralateral VPM (**C**) of naïve (*n* = 8), sham (*n* = 7), and SNL animals (*n* = 8) collected 14 days after the beginning of the experiments, with high expression level appearing in yellow and low expression level in dark blue. Red arrows indicate genes for which mRNA expression is significantly altered by the experimental conditions. For these genes, the mean fold change is presented on bar histograms. In the contralateral VPL, mRNA expression of the CTSS gene (**A2**: one-way ANOVA *F*(2, 13) = 8.103, *p* = 0.0052) and of the CX3CR1 gene (**A3**: one-way ANOVA *F*(2, 13) = 4.043, *p* = 0.0431) is significantly increased in SNL animals compared to naïve rats. The expression of these two genes is correlated to the ambulatory pain. The less weight the animal bears on its ipsilateral hind paw, the higher the mRNA expression of the CTSS (**A4**) and the CX3CR1 (**A5**) genes is. No significant difference was observed in the mRNA expression of the tested genes in the ipsilateral VPL (**B**) and contralateral VPM (**C**). It must be noted that, due to technical problems, only five genes were tested on the contralateral VPM. Post hoc test (Tukey’s multiple comparison test): *p < 0.05; **p < 0.01

We performed Q-PCR on microdissected contralateral (Fig. 3A1) and ipsilateral (Fig. 3B) VPL as well as contralateral VPM (Fig. 3C) of naïve, sham, and SNL animals collected 14 days after the beginning of the experiment. The amount of tissue collected allowed exploring the mRNA expression of nine genes (see Table 1). We found that in the contralateral VPL, mRNA expression of the CTSS gene (Fig. 3A2) and of the C×3CR1 gene (Fig. 3A3) was significantly increased in SNL animals compared to naïve rats (1.3 times greater). Moreover, the expression of these two genes was correlated to the ambulatory pain. The less weight the animal bore on its ipsilateral hind paw, the higher the mRNA expression of the CTSS (Fig. 3A4) and the CX3CR1 (Fig. 3A5) genes was. No significant difference was observed in the mRNA expression of the tested genes in the ipsilateral VPL (Fig. 3B) and contralateral VPM (Fig. 3C). It should be noted that, due to low amount of mRNA collected from VPM, only five genes were tested on the microdissected VPM tissues.

**Fig. 4:**
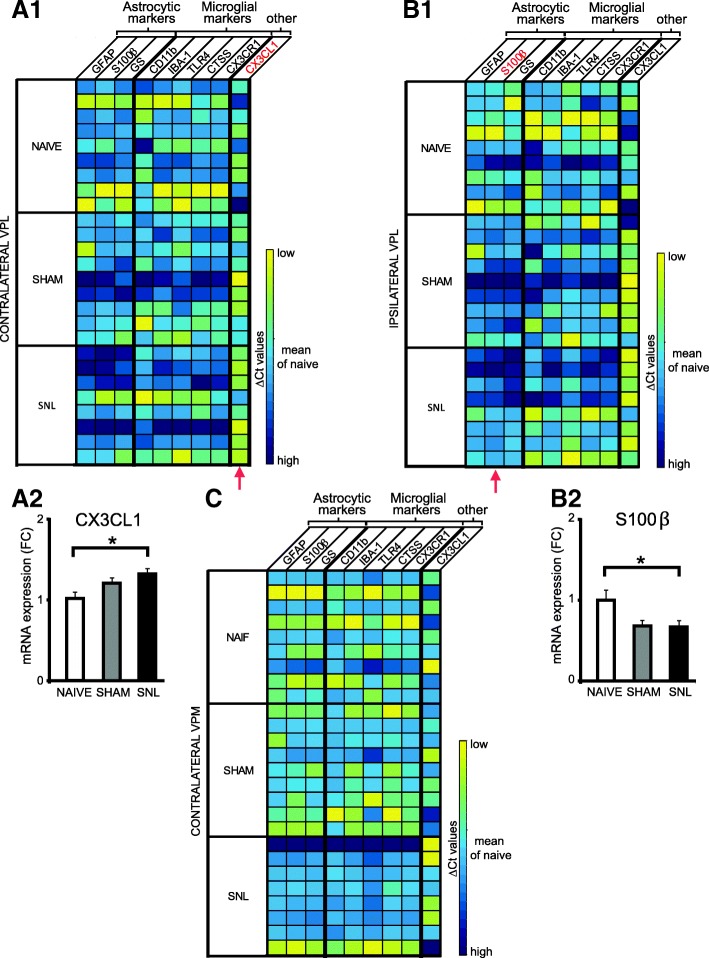
Altered mRNA expression in the microdissected VPL 28 days after spinal nerve ligation. Tables summarize the expression of up to nine genes on microdissected contralateral (**A1**) and ipsilateral (**B1**) VPL as well as contralateral VPM (**C**) of naïve (*n* = 9), sham (*n* = 9), and SNL animals (*n* = 8) collected 28 days after the beginning of the experiments, with high expression level appearing in yellow and low expression level in dark blue. Red arrows indicate genes for which mRNA expression is significantly altered by the experimental conditions. In the contralateral VPL, mRNA expression of the CX3CL1 gene is significantly increased in SNL animals compared to naïve rats (**A2**: one-way ANOVA *F*(2, 23) = 4.243, *p* = 0.027) while in the ipsilateral VPL mRNA of the S100β gene is significantly decreased in SNL animals compared to naïve rats (**B2**: one-way ANOVA *F*(2, 23) = 3.972, *p* = 0.033). Even though mRNA expression of the tested genes appears low in the contralateral VPM of SNL animals (**C**), no significant difference is found. Post hoc test (Tukey’s multiple comparison test): *p < 0.05

We also performed Q-PCR on microdissected contralateral (Fig. 4A1) and ipsilateral (Fig. 4B1) VPL as well as contralateral VPM (Fig. 4C) of naïve, sham, and SNL animals collected 28 days after the beginning of the experiment. We found that in the contralateral VPL, mRNA expression of the C×3CL1 gene was significantly increased in SNL animals compared to naïve rats (nearly 1.3 times greater; Fig. 4A2), while in the ipsilateral VPL, mRNA of the S100β gene was significantly decreased in SNL animals compared to naïve rats (33% decrease; Fig. 4B2; surgery alone (sham animals) also tended to decrease mRNA of the S100β gene compared to naïve rats, post hoc test: *p* = 0.054).

In conclusion, we found a specific increase in mRNA expression of cathepsin S and fractalkine receptor 14 days after the beginning of the experiment and then a specific increase in mRNA expression of fractalkine 28 days after the beginning of the experiment within the laser microdissected contralateral VPL of SNL animals compared to naïve animals (see summary Fig. 12). These are well-known markers of microglial reactivity.

### Altered fractalkine receptor protein levels 14 days after spinal nerve ligation

**Fig. 5:**
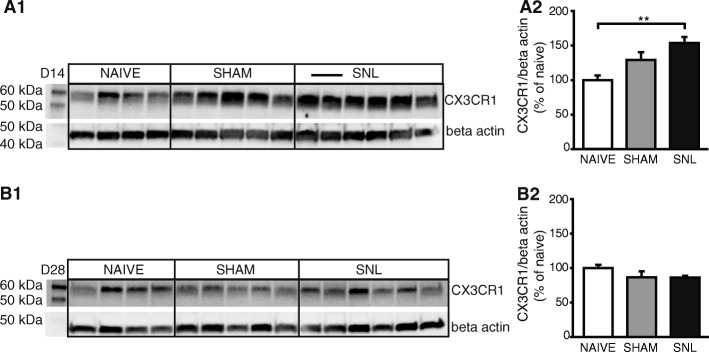
Increased fractalkine receptor protein level in the contralateral thalamus only 14 days after spinal nerve ligation. Western blot (**A1** and **B1**) and densitometric analysis (**A2** and **B2**) were performed on contralateral thalamic protein extracts from naïve (*n* = 4), sham (*n* = 5), and SNL (*n* = 6) animals 14 (**A1** and **A2**) or 28 (**B1** and **B2**) days after the beginning of the experiments. CX3CR1 (fractalkine receptor) protein level is significantly increased in SNL animals compared to naïve rats 14 days after the beginning of the experiment (**A2**: one-way ANOVA *F*(2, 12) = 7.877, *p* = 0.0065) while no significant difference is found 28 days after the beginning of the experiment (**B2**: one-way ANOVA *F*(2, 12) = 1.645, *p* = 0.2336). Post hoc test (Tukey’s multiple comparison test): **p < 0.01

To determine whether altered mRNA expression resulted in altered protein expression, we examined protein expression by Western blotting extracts from formalin-fixed lateral thalamus including the cVPL of naïve, sham, and SNL rats collected 14 or 28 days after the beginning of the experiment. The CX3CR1 antiserum recognized a single band with an apparent molecular weight of 55 kDa (Fig. 5A1, B1). Western blot quantification revealed that at D14, CX3CR1 protein level in the contralateral thalamus was significantly increased in SNL animals compared to naïve rats (1.5 times greater; Fig. 5A1, A2) while no significant difference was observed in the CX3CR1 protein level at D28 (Fig. 5B1, B2).

In conclusion, in addition to the specific increase in mRNA expression of the fractalkine receptor, we also found an increase in the fractalkine receptor protein level in the contralateral thalamus of SNL animals compared to naïve animals 14 days after the beginning of the experiment.

### Decreased iba-1 immunostaining and decreased number of iba-1 positive cells in the VPL 14 days after spinal nerve ligation

**Fig. 6:**
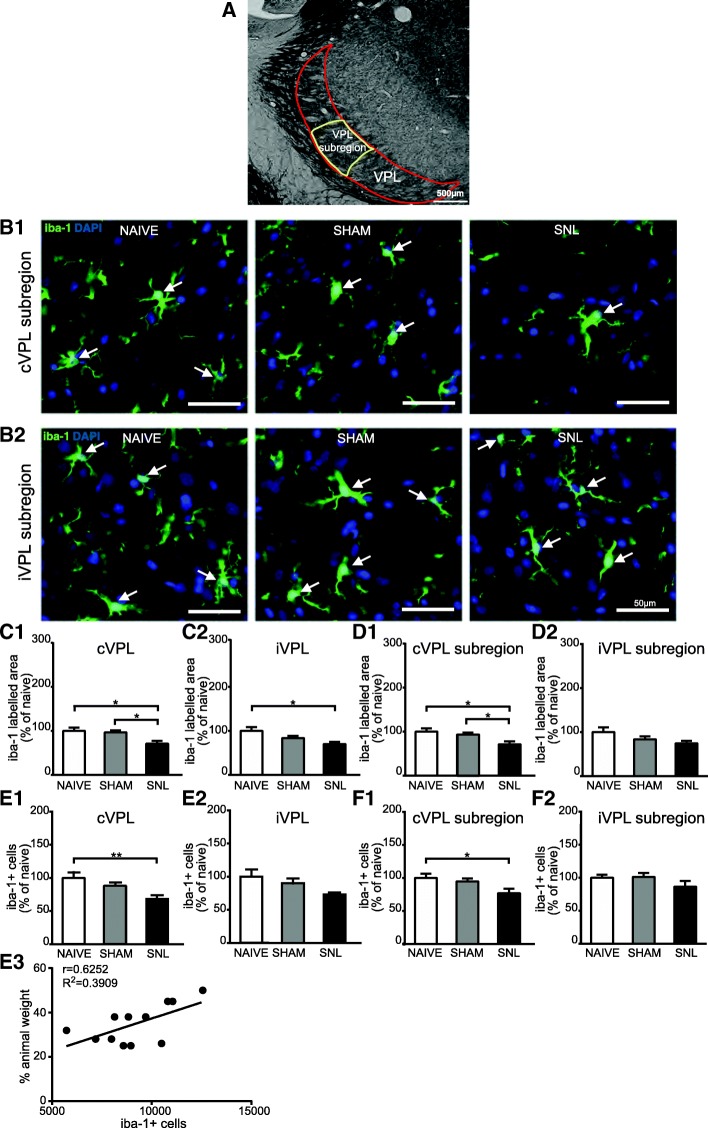
Decreased iba-1 immunostaining and decreased number of iba-1-positive cells in the contralateral VPL 14 days after spinal nerve ligation. Panel **A** illustrates the delineation of the ventral posterolateral thalamic nucleus (VPL; outlined in red) with its subregion that receives information from the hind limb (VPL subregion; outlined in yellow) on a rat brain section treated for visualizing acetylcholinesterase activity. The outlines were then applied on an adjacent section treated for the immunohistofluorescent detection of the iba1. Microphotographs in **B1** and **B2** are examples of iba-1 immunostaining (green) and DAPI staining (deep blue) in the contralateral (cVPL, **B1**) and ipsilateral (iVPL, **B2**) subregion of the VPL in naïve, sham, and SNL animals 14 days after the beginning of the experiments. White arrows point at iba-1/DAPI-positive cells (part of the cell body appears in turquoise, that is to say mix of green and deep blue). Note that there is less iba-1 immunostaining and less iba-1/DAPI-positive cells in the cVPL subregion of SNL rats. Morphometric analysis conducted on the VPL (**C1** and **C2**) and on the VPL subregion (**D1** and **D2**) reveals that iba-1 immunofluorescent surfaces (expressed as percentage of naïve values) are significantly decreased in the cVPL (**C1**: one-way ANOVA *F*(2, 13) = 5.151, *p* = 0.0225) as well as in the cVPL sub-region (**D1**: one-way ANOVA *F*(2, 13) = 4.716, *p* = 0.0288) in the SNL rats (*n* = 6, 3 slices per animal) compared to naïve (*n* = 4, 3 slices per animal) and sham (*n* = 6, 3 slices per animal) rats. This decrease is also found in on the ipsilateral side (**C2** and **D2**) but only reaches statistical significance in the iVPL of SNL rats compared to naïve rats (**C2**: one-way ANOVA *F*(2, 13) = 4.183, *p* = 0.0396). Quantification of iba-1/DAPI-positive cells in the VPL (**E1** and **E2**; cell number per volume unit expressed as percentage of naïve values) by using an optical disector method of stereological counting and in the VPL subregion by using a conventional method (**F1** and **F2**; cell number per surface area unit expressed as percentage of naïve values) reveals that the number of iba-1/DAPI-positive cells is significantly reduced in the cVPL (**E1**: one-way ANOVA *F*(2, 13) = 6.669, *p* = 0.0102) as well as in the cVPL subregion (**F1**: one-way ANOVA *F*(2, 13) = 4.679, *p* = 0.0295) of SNL rats compared to the one of naïve rats. No significant difference is found in the iVPL (**E2**: one-way ANOVA *F*(2, 13) = 3.784, *p* = 0.0507) and the iVPL subregion (**F2**: one-way ANOVA *F*(2, 13) = 2.878, *p* = 0.0923). The number of iba-1/DAPI-positive cells in the cVPL is correlated to the ambulatory pain. The less weight the animal bears on its ipsilateral hind paw, the lower the number of iba-1/DAPI positive cells is (**E3**). Post hoc test (Tukey’s multiple comparison test): *p < 0.05; **p < 0.01

To further determine whether spinal nerve ligation leads to microglia activation in the thalamus that receives projections from ascending pain pathways, we used morphometric analysis of iba-1 immunostaining as well as iba-1/DAPI-positive cell counts. Representative microphotographs of iba-1 immunostaining in the VPL (delineated in Fig. 6A) of naïve, sham, and SNL animals 14 days after the beginning of the experiments are displayed in Fig. 6B1 and B2. Morphometric analysis of the iba-1-immunolabeled surface was performed in the entire VPL (Fig. 6C1 and C2), but also more specifically in the VPL subregion (Fig. 6D1 and D2) that receives information from the hindlimb (delineated in Fig. 6A). This analysis led to the same results in the contralateral VPL and contralateral VPL subregion. Indeed, in both regions, iba-1 immunostaining occupied a significantly smaller area in SNL animals than in naïve and sham rats (35% decrease; Fig. 6B1, C1, and D1). This tendency was also found in the ipsilateral VPL (Fig. 6C2) and VPL subregion (Fig. 6D2), but iba-1-immunostained surface was only significantly smaller in the ipsilateral VPL of SNL rats compared to the one of naïve rats (Fig. 6C2). By performing stereological counting in the whole VPL, we found that iba-1/DAPI-positive cell density was significantly lower in SNL animals compared to naïve animals on the contralateral side (31.5% decrease; Fig. 6E1). Furthermore, iba-1/DAPI-positive cell density was correlated to ambulatory pain. Indeed, the less weight the animal bore on its ipsilateral hind paw, the lower the density of iba-1/DAPI-positive cells was in the contralateral VPL (Fig. 6E3). By using conventional counts in the VPL subregion, we found that the iba-1/DAPI-positive cell number per surface area unit was significantly lower in SNL animals compared to naïve animals on the contralateral side (21% decrease; Fig. 6F1). Both counting methods revealed that the experimental conditions did not significantly alter the iba-1/DAPI-positive cell number in the ipsilateral VPL (Fig. 6E2) and ipsilateral VPL subregion (Fig. 6F2). Therefore, both counting methods led to the same results.

**Fig. 7:**
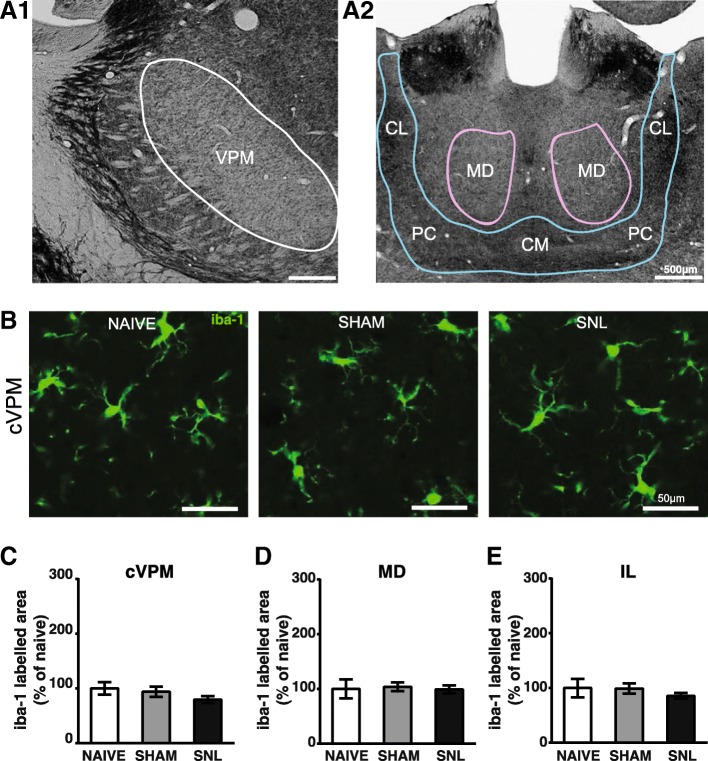
Unaltered iba-1 immunostaining in the contralateral VPM (cVPM) and MD and intralaminar thalamic nuclei (IL) 14 days after spinal nerve ligation. Microphotographs in **A1** and **A2** illustrate the delineation of the ventral posteromedian thalamic nucleus (VPM; outlined in white in **A1**) and the mediodorsal thalamic nucleus (MD; outlined in purple in **A2**) and the group of the intralaminar thalamic nuclei (outlined in blue in **A2**) that includes the central medial thalamic nucleus (CM), the paracentral thalamic nucleus (PC), and the central lateral thalamic nucleus (CL) on a rat brain section treated for visualizing acetylcholinesterase activity. The outlines were then applied on an adjacent section treated for the immunohistofluorescent detection of the iba1. Microphotographs in **B** are examples of iba-1 immunostaining (green) in the cVPM of naïve, sham, and SNL animals 14 days after the beginning of the experiments. Morphometric analysis (SNL and sham: *n* = 6; naïve: *n* = 4; 3 slices per animal) reveals that the treatment does not alter the iba-1 immunofluorescent surfaces in the cVPM (**C**: one-way ANOVA *F*(2, 13) = 4.244, *p* = 0.0381 but no significant differences with the Tukey’s multiple comparison test), MD (**D**: one-way ANOVA *F*(2, 13) = 0.07, *p* = 0.9322), and IL (**E**: one-way ANOVA *F*(2, 13) = 0.6494, *p* = 0.5385)

Changes in iba-1-immunostained surface upon experimental conditions were not found 14 days after the beginning of the experiments in the contralateral VPM (delineation: Fig. 7A1; representative pictures: Fig. 7B; quantification: Fig. 7C), in the MD (delineation: Fig. 7A2; quantification: Fig. 7D), and in the IL (delineation: Fig. 7A2; quantification: Fig. 7E). Indeed no difference was found between experimental conditions.

**Fig. 8:**
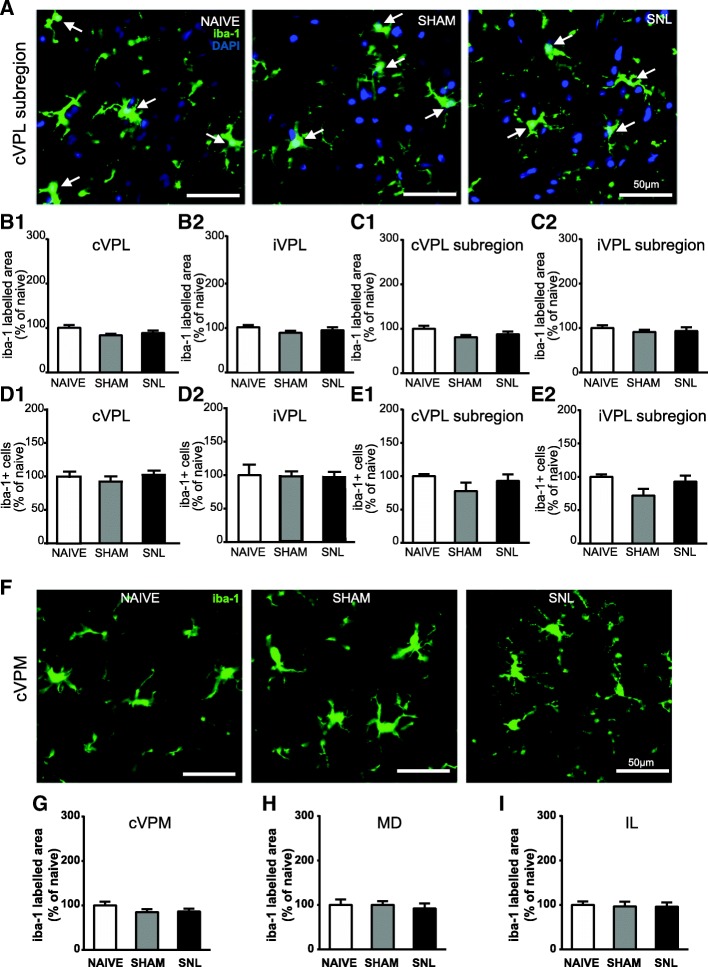
Unaltered iba-1 immunostaining in the VPL, cVPM, MD, and IL as well as unaltered number of iba-1-positive cells in the VPL 28 days after spinal nerve ligation. Microphotographs are examples of iba-1 immunostaining (green) and DAPI staining (deep blue) in the cVPL (**A**) in naïve, sham, and SNL animals 28 days after the beginning of the experiments. White arrows point at iba-1/DAPI-positive cells (part of the cell body appears in turquoise, that is to say a mix of green and deep blue). Morphometric analysis conducted on the VPL (contralateral, **B1**: one-way ANOVA *F*(2, 11) = 0.489, *p* = 0.626; ipsilateral, **B2**: one-way ANOVA *F*(2, 11) = 0.7396, *p* = 0.4996) and on the VPL subregion (contralateral, **C1**: one-way ANOVA *F*(2, 11) = 0.3216, *p* = 0.7316; ipsilateral, **C2**: one-way ANOVA *F*(2, 11) = 0.6745, *p* = 0.5293) reveals that iba-1 immunofluorescent surfaces are unchanged under treatment (naïve and SNL: *n* = 5, sham: *n* = 4, 3 slices per animal). Quantification of iba-1/DAPI-positive cells in the VPL (contralateral, **D1**: one-way ANOVA *F*(2, 11) = 0.5755, *p* = 0.5785; ipsilateral, **D2**: one-way ANOVA *F*(2, 11) = 0.1196, *p* = 0.8884) by using an optical disector method of stereological counting (cell number per volume unit) and in the VPL subregion (contralateral, **E1**: one-way ANOVA *F*(2, 11) = 0.4605, *p* = 0.6426; ipsilateral, **E2**: one-way ANOVA *F*(2, 11) = 3.934, *p* = 0.0514) by using a conventional method (cell number per surface area unit) reveals no significant difference between experimental conditions. Microphotographs in **F** are examples of iba-1 immunostaining (green) in the cVPM in naïve, sham, and SNL animals twenty eight days after the beginning of the experiments. Morphometric analysis reveals that the treatment does not alter the iba-1 immunofluorescent surfaces in the cVPM (**G**: one-way ANOVA *F*(2, 11) = 0.04787, *p* = 0.9535), MD (**H**: one-way ANOVA *F*(2, 11) = 0.1803, *p* = 0.8375), and IL (**I**: one-way ANOVA *F*(2, 11) = 0.05713, *p* = 0.9447)

Twenty eight days after the beginning of the experiments, iba-1-immunostained area (representative pictures in Fig. 8A) was not altered upon experimental conditions in the contralateral (Fig. 8B1) and ipsilateral (Fig. 8B2) VPL and in the contralateral (Fig. 8C1) and ipsilateral (Fig. 8C2) VPL subregion. Likewise, iba-1/DAPI-positive cell number per volume unit was unchanged upon experimental conditions in the contralateral (Fig. 8D1) and ipsilateral (Fig. 8D2) VPL, and iba-1/DAPI-positive cell number per surface area unit was unchanged upon experimental conditions in the contralateral (Fig. 8E1) and ipsilateral (Fig. 8E2) VPL subregion. In the same way, iba-1-immunostained surface remained unchanged upon experimental conditions 28 days after the beginning of the experiments in the contralateral VPM (representative pictures: Fig. 8F; quantification: Fig. 8G), in the MD (Fig. 8H), and in the IL (Fig. 8I).

In conclusion, we found that the iba-1 immunostaining area and the number of iba-1 immunopositive cells were specifically decreased in the contralateral VPL of SNL as compared to the one of naïve rats 14 days after the beginning of the experiment (see summary Fig. 12).

### Increased GFAP immunostaining in the thalamus 28 days after spinal nerve ligation

To determine whether spinal nerve ligation leads to astrocytic activation in the thalamus that receives projections from ascending pain pathways, we used morphometric analysis of GFAP immunostaining as well as GFAP/S100β/DAPI-positive cell counts. Both stereological counting and conventional counts led to the same results for the iba-1-positive cells 14 and 28 days after the beginning of the experiments. Consequently, we only performed conventional counts in the VPL subregion for the GFAP-S100β-positive cells.

**Fig. 9:**
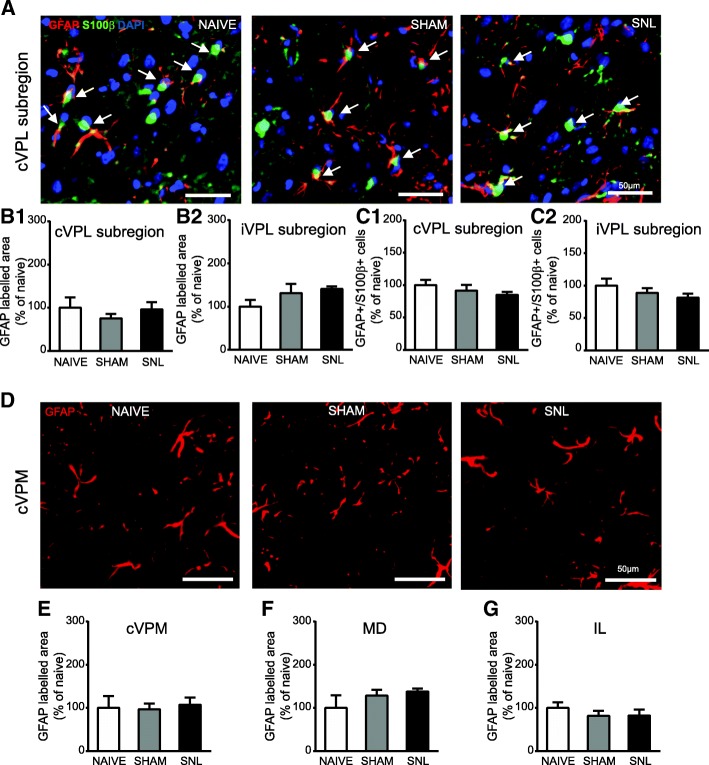
Unaltered GFAP immunostaining in the VPL, cVPM, MD, and IL and unaltered number of GFAP/S100β-positive cells in the VPL 14 days after spinal nerve ligation. Microphotographs in **A** are examples of GFAP (red) and S100β (green) immunostaining as well as DAPI staining (deep blue) in the cVPL subregion in naïve, sham, and SNL animals 14 days after the beginning of the experiments. White arrows point at GFAP/S100β/DAPI-positive cells (part of the cell body appears in turquoise (green and deep blue) and is closed to at least two thick GFAP-labeled fibers). Morphometric analysis conducted on the contralateral (**B1**: Kruskal-Wallis test *H*(2) = 1.256, *p* = 0.5337) and ipsilateral (**B2**: one-way ANOVA *F*(2, 12) = 1.906, *p* = 0.1911) VPL subregion reveals that GFAP immunofluorescent surfaces are unchanged under treatment (naïve: *n* = 4; sham: *n* = 5; SNL: *n* = 6; 3 slices per animal). Quantification of GFAP/S100β/DAPI-positive cells in the contralateral (**C1**: one-way ANOVA *F*(2, 12) = 1.402, *p* = 0.2838) and ipsilateral (**C2**: one-way ANOVA *F*(2, 12) = 1.087, *p* = 0.3684) VPL subregion reveals no significant difference between experimental conditions. Microphotographs in **D** are examples of GFAP immunostaining (red) in the cVPM of naïve, sham, and SNL animals 14 days after the beginning of the experiments. Morphometric analysis reveals that the treatment does not alter the GFAP immunofluorescent surfaces in the cVPM (**E**: Kruskal-Wallis test *H*(2) = 1.148, *p* = 0.5634), MD (**F**: one-way ANOVA *F*(2, 12) = 1.384, *p* = 0.2879), and IL (**G**: one-way ANOVA *F*(2, 12) = 0.5574, *p* = 0.5869)

Representative microphotographs of GFAP and S100β immunostaining in the contralateral VPL subregion of naïve, sham, and SNL animals 14 days after the beginning of the experiments are displayed in Fig. 9A. Morphometric analysis revealed that the GFAP-immunolabeled area remained unchanged upon experimental conditions in the contralateral (Fig. 9B1) and ipsilateral (Fig. 9B2) VPL subregion. Same results were obtained when the analysis was performed in the whole VPL (data not shown). Likewise, the GFAP/S100β/DAPI-positive cell number was unaffected by the experimental conditions in the contralateral (Fig. 9C1) and ipsilateral (Fig. 9C2) VPL subregion. Similarly, GFAP-immunostained surface remained unchanged upon experimental conditions 14 days after the beginning of the experiments in the contralateral VPM (representative pictures: Fig. 9D; quantification: Fig. 9E), in the MD (Fig. 9F), and in the IL (Fig. 9G).

**Fig. 10:**
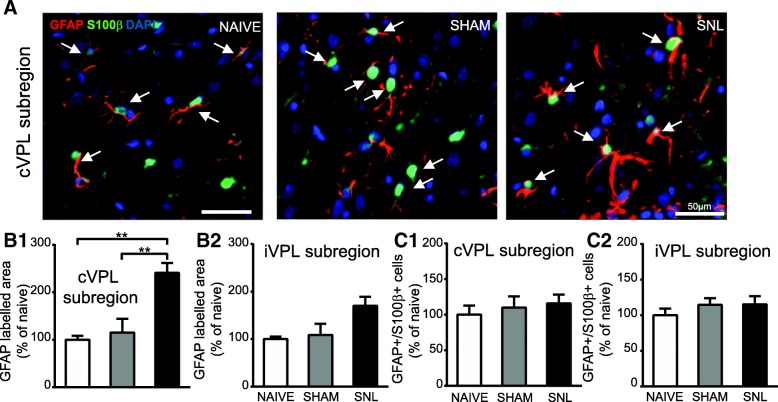
Increased GFAP immunostaining and unaltered number of GFAP/S100β-positive cells in the VPL 28 days after spinal nerve ligation. Microphotographs are examples of GFAP (red) and S100β (green) immunostaining as well as DAPI staining (deep blue) in the cVPL subregion (**A**) in naïve, sham, and SNL animals 28 days after the beginning of the experiments. White arrows point at GFAP/S100β/DAPI-positive cells (part of the cell body appears in turquoise (green and deep blue) and is closed to at least two thick GFAP-labeled fibers). Note that the GFAP-labeled fibers are thicker in the cVPL subregion of SNL rats but that there is the same amount of GFAP/S100β/DAPI-positive cells. Morphometric analysis reveals that GFAP immunofluorescent surfaces are significantly increased in the cVPL subregion (**B1**: one-way ANOVA *F*(2, 12) = 12.62, *p* = 0.0011) of SNL rats (*n* = 6, 3 slices per animal) compared to the ones of naïve (*n* = 4, 3 slices per animal) and sham (*n* = 5, 3 slices per animal) rats but that is not the case in the iVPL subregion (**B2**: one-way ANOVA *F*(2, 12) = 4.246, *p* = 0.0403, but no significant differences with the Tukey’s multiple comparison test). Quantification of GFAP/S100β/DAPI-positive cells in the contralateral (**C1**: one-way ANOVA *F*(2, 12) = 0.5752, *p* = 0.5763) and ipsilateral (**C2**: one-way ANOVA *F*(2, 12) = 0.285, *p* = 0.7566) VPL subregion reveals no significant difference between experimental conditions. Post hoc test (Tukey’s multiple comparison test): **p < 0.01

**Fig. 11:**
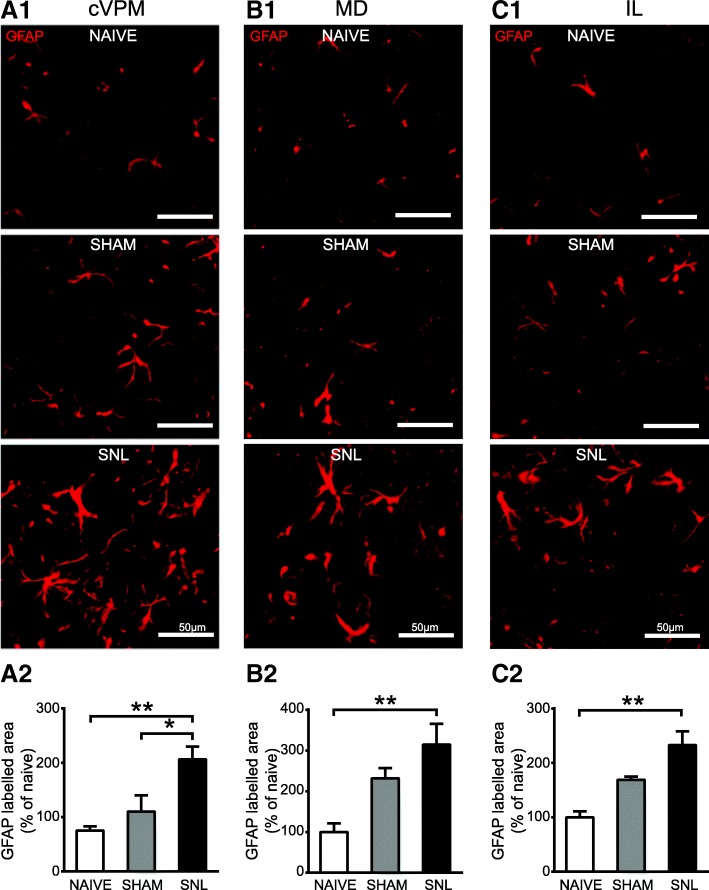
Increased GFAP immunostaining in the contralateral VPM (cVPM) and MD and IL 28 days after spinal nerve ligation. Microphotographs are examples of GFAP immunostaining (red) in the cVPM (**A1**), MD (**B1**), and IL (**C1**) in naïve, sham, and SNL animals 28 days after the beginning of the experiments. Note that GFAP immunostaining is increased in each thalamic nucleus of the SNL rats. Morphometric analysis reveals that GFAP immunofluorescent surfaces are significantly increased in each thalamic nuclei of SNL rats (*n* = 6, 3 slices per animal) compared to the ones of naïve (*n* = 4, 3 slices per animal) rats (**A2**: one-way ANOVA *F*(2, 12) = 8.102, *p* = 0.0059; **B2**: one-way ANOVA *F*(2, 12) = 6.856, *p* = 0.0103; **C2**: one-way ANOVA *F*(2, 12) = 11.87, *p* = 0.0014) and that GFAP immunofluorescent surfaces are significantly increased in the cVPM of SNL rats compared to the ones of sham (*n* = 5, 3 slices per animal) rats (**A**2). Post hoc test (Tukey’s multiple comparison test): *p < 0.05; **p < 0.01

By contrast, 28 days after the beginning of the experiments, the GFAP-immunostained area was significantly increased in the contralateral VPL subregion of SNL animals compared to naïve and sham rats (nearly 2.5 times greater; representative pictures in Fig. 10A and quantification in Fig. 10B1). Even though a similar tendency was found in the ipsilateral VPL subregion, this did not reach a statistical significance (Fig. 10B2). However, GFAP/S100β/DAPI-positive cell number remained unchanged upon experimental conditions in the contralateral (Fig. 10C1) and ipsilateral (Fig. 10C2) VPL subregion. A significant increase in GFAP-immunostained area 28 days after the beginning of the experiments was also found in the contralateral VPM (2.75 times greater; representative pictures: Fig. 11A1; quantification: Fig. 11A2), in the MD (3.1 times greater; representative pictures: Fig. 11B1; quantification: Fig. 11B2), and in the IL (2.3 times greater; representative pictures: Fig. 11C1; quantification: Fig. 11C2) of SNL animals compared to naïve rats. In the contralateral VPM, GFAP-immunostained area was also significantly higher in SNL rats compared to sham rats (1.9 times greater; Fig. 11A2).

**Fig. 12:**
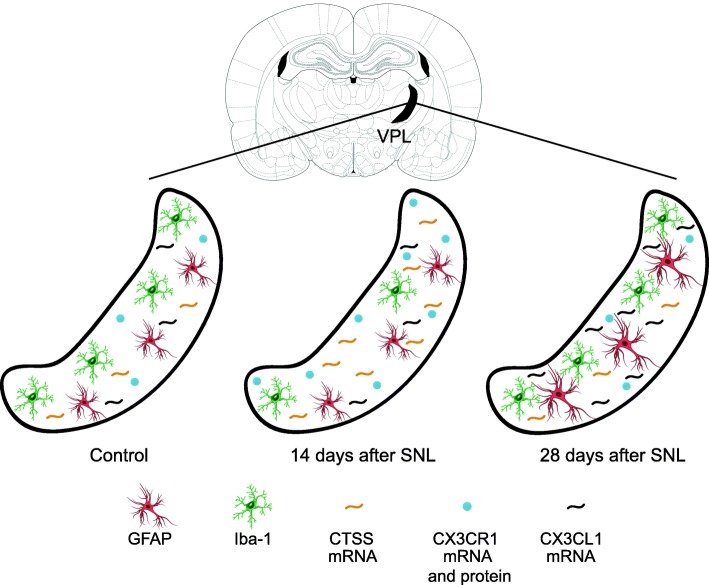
Schematic summarizing sequential alteration in astrocytes and microglia in the VPL 14 and 28 days after spinal nerve ligation (SNL). The VPL is delineated in black on Figure 56 (interaural 6.24 mm, Bregma − 2.76 mm) from the atlas of Paxinos and Watson [48]. Schemas of astrocytes appear in red (GFAP immunolabeling) and schemas of microglia (iba-1immunolabelling) appear in green. We found that GFAP immunostaining area was increased at D28 in the VPL of SNL as compared to the one of naïve rats while the number of GFAP/S100β immunopositive cells was unchanged. At D14, the iba-1 immunostaining area and the number of iba-1 immunopositive cells were decreased in the VPL of SNL as compared to the one of naïve rats. Finally, we found a sequential increase in mRNA expression of cathepsin S (D14), fractalkine (CX3CL1, D28), and fractalkine receptor (CX3CR1, D14, also increase in protein expression), well-known markers of microglial reactivity

In conclusion, we found that GFAP immunostaining area was broadly increased in thalamic nuclei of SNL animals as compared to the one of naïve rats 28 days after the beginning of the experiment. In the VPL, this was not accompanied by an increased number of GFAP/S100β immunopositive cells (see summary Fig. 12).

## Discussion

The main goal of this study was to investigate the involvement of thalamic astrocytes and microglia in the onset and maintenance of neuropathic pain. By correlating pain behavior with changes in astrocytes and microglia morphology, number and molecular repertoire 2 and 4 weeks after a unilateral L5/L6 spinal nerve ligation, we found an early transient and spatially restricted microglial alteration in VPL followed by a late and widespread astrocytic activation in the thalamus of SNL animals (Fig. 12).

### New insights in SNL pain behavior

We found that SNL animals developed static mechanical allodynia and hyperalgesia as well as reduction in body weight borne on the ligated limb. These symptoms were accompanied by a redistribution of the body load on the unoperated hind limb as well as the front limbs. In addition to these classical pain symptoms, we revealed a previously undescribed compensatory mechanism. Indeed, a transient pronounced increase of pain threshold was observed on the unoperated hind limb of SNL animals 2 weeks after surgery. As it has been suggested that the mechanism involved in the onset of neuropathic pain may differ from those involved in maintenance [10, 20, 22], it is tempting to speculate that, in our model, a compensatory mechanism linked/related to the initiation of neuropathic pain takes place to decrease the pain sensitivity of this limb. Once the switch has been operated between initiation and maintenance, this compensatory hypoalgic behavior would disappear.

Since the initial description of the SNL model [28], SNL and sham groups were mainly used in the literature. As sham animals underwent surgery including removal of L6 transverse process and isolation of L5 and L6 spinal nerves, we also included naïve rats in order to assess the impact of the surgery on the pain behavior. Indeed, it is well known that incision that includes skin and muscles is able to cause a mechanical hyperalgesia lasting for up to 3 days after surgery [49]. In our study, we found a transient mechanical static hyperalgesia on the operated hind paw of sham rats 2 weeks after the beginning of the experiments. Therefore, the hyperalgesia lasts longer than following a simple incision. Indeed, our result is in agreement with the study of Kosta and collaborators in which they described a hyperalgesia in animals 2 weeks after a L5-S1 laminectomy including removal of L6 transverse and L5-L6 spinous processes without ganglionectomy [50]. Therefore, we show that, in addition to incision, removal of L6 transverse process is able to induce a static mechanical hyperalgesia that is no longer present 4 weeks after the surgery. It should be noted that we could not detect any allodynia or ambulatory pain in sham animals compared to naïve, arguing for the development of a moderate pain in sham animals. However, the fact that sham animals display mechanical hyperalgesia at day 14 suggests that potential glial impairment resulting from surgical procedures (i.e., even skin and muscle incisions are able to induce activation of spinal astrocytes and microglia [51]) may preclude the detection of further changes due to spinal nerve ligation. This may be further amplified by the fact that individual marker of glial impairment may reach their expression plateau at different levels of pain severity.

### Complex microglial alteration in the VPL of SNL animals

Concerning the microglia, our immunohistochemical results show that the number of iba-1 immunopositive cells and the iba-1-immunostained area are specifically decreased in the VPL 2 weeks after the spinal nerve ligation. These results are particularly interesting, as they go against some previously published evidence. Indeed, it has been shown that mRNA expression or immunoreactivity for some microglial markers is, on the contrary, increased in the VPL following sciatic nerve transection [27] or chronic constriction injury of the sciatic nerve [25, 52, 53]. It is important to note that microglial activation was found in the thalamus 14, 16, 28, or 34 days after the peripheral nerve injury compared to naïve [27, 52] or sham animals [53]. Furthermore, a study examining patients suffering from lumbar chronic pain since at least 2 years has reported evidence for microglial activation in the thalamus [54]. Therefore, even if we could not definitively rule out the possibility that we missed an early time point of microglial activation at the thalamic level by assessing glial reactivity at 2 and 4 weeks after the spinal nerve ligation, our results clearly provide evidence for a new type of VPL microglial alteration in neuropathic condition. We are confident in our results for three main methodological reasons: (1) cells were counted using two distinct methods (conventional count and an optical dissector method) that lead to the same results, (2) the decreased number of microglial cells was found within the VPL as well as within the subregion of the VPL that receives nociceptive information from the operated hind limb, and (3) the decreased number of microglial cells was paralleled by the decreased iba-1-immunostained area. Furthermore, the decreased iba-1-immunostained area was specifically found in the VPL (not in the VPM, DM, and IL) suggesting that this microglial alteration was not generalized to the whole thalamus. Finally and more interestingly, the number of microglial cells was negatively correlated with the ambulatory pain, suggesting a possible link between the number of microglial cells and pain symptoms.

Our results raise intriguing issues. For instance, the decreased microglial immunoreactivity is not found any more after 4 weeks of spinal nerve ligation. Such a transient decrease in microglial cell number may be explained if we postulate (i) a transient downregulation of iba-1 expression (below the immunohistochemichal detection level) 2 weeks after surgery, (ii) a sequence of apoptosis followed by gliogenesis, or (iii) a migration of microglia. Downregulation of iba-1 appears unlikely as we found no change in iba-1 mRNA expression. Apoptosis of microglial cells appears also unlikely as pilot experiments using caspase-3 immunodetection did not show any obvious sign of apoptosis in the VPL of SNL animals (data not shown). The last possibility was not explored. Another important issue is that, while no sign of microglial activation (such as hypertrophy and increased cell number) was found using immunohistochemical methods, we detected molecular signs of microglial activation. Indeed, we found that mRNA expression of, on one hand, CTSS and the receptor of fractalkine (CX3CR1) and, on the other hand, fractalkine (CX3CL1) was increased in the VPL 2 weeks and 4 weeks respectively after spinal nerve ligation. Furthermore, the expression of CTSS and CX3CR1 gene was positively correlated to the ambulatory pain. Finally, the CX3CR1 protein expression was also increased in the VPL 2 weeks after spinal nerve ligation. At this point, it must be noted that all these above reported differences evidenced by qRT-PCR and Western blot were only statistically significant between naïve and SNL animals, the values of sham animals being intermediate. At this stage, we cannot exclude that the differences observed between naïve and SNL animals may in part result from surgical procedures in addition to spinal nerve ligation. The involvement of the fractalkine/CTSS system in the amplification and maintenance of neuropathic pain has been extensively studied in spinal cord [17, 41–43, 55–57]. However, whether this system is also involved in modulating pain perception in thalamic nuclei remains totally unexplored. Nevertheless, our results demonstrate upregulation of mRNA (cathepsin S, CX3CR1, and CX3CL1) and protein (CX3CR1) expression of key players of the neuron-microglia signaling pathway that is critically involved in neuropathic pain. We are thus facing a novel and complex situation in which we get molecular signs of microglial activation while, at the morphological level, microglia present absolutely no sign of activation even being, at the opposite, less dense. Microglial decline has been reported following exposure to chronic unpredictable stress in mice, but this occurs following an initial period of microglial activation [58]. Indeed, this microglial decline has been interpreted as senescence of previously intensely and lastingly activated microglia leading to dysfunctional microglia that may have detrimental effects on synapse structure and functioning, neurogenesis and neurotrophines action. Therefore, to the best of our knowledge, our concomitant signs of microglial activation and decline are unique and may represent an unclassical model of microglial alteration.

### Astrocytic activation in the thalamus of SNL animals

Regarding the astrocytes, we found, 4 weeks after the spinal nerve ligation, an increase in GFAP immunoreactivity in the VPL with no change in astrocyte number, leading to the conclusion that the astrocytes were hypertrophied (compare astrocytes in left and right microphotographs in Fig. 10A). This hypertrophy is a sign of astrocytic activation [59]. It has been shown in the mice hippocampus that all GFAP-positive cells are also S100β positive [60]. Therefore, our cell counts made on GFAP/S100β/DAPI-positive cells are likely to represent a real estimation of the number of GFAP-positive cells and not an underestimate. Astrocytic activation is known to be accompanied by an upregulation of GFAP mRNA [59]. This is not the case in our model since we even report a decreased S100β mRNA expression in the ipsilateral VPL 4 weeks after the spinal nerve ligation. Therefore, the delay after the surgery may have been insufficient to get a full astrocytic activation or conversely astrocytic activation may be at a point where it is decreasing. Beyond this, no sign of thalamic astrocytic activation has been reported 10 days after spared nerve injury [61] nor 7 to 14 days after chronic constriction injury of the sciatic nerve [25] while Giardini et al. [52] observed an increase in GFAP protein levels in the thalamus 34 days after CCI. Therefore, it could be that ligation of peripheral nerves induces a delayed thalamic astrocytic activation.

It must be noted that the astrocytic activation was not restricted to the VPL but was also found in the VPM, the DM, and the IL, suggesting a widespread response of the thalamus involved in pain processing including the sensory discriminative aspects (VPL and VPM) as well as the affective components (DM and IL). While the VPM receives facial sensory information, Wu et al. have already reported glial alteration in the VPM without nerve lesions in the orofacial area as they performed a thoracic spinal cord injury [62].

Upon activation, astrocytes can release pro-inflammatory factors like cytokines, prostaglandins, neurotrophins, and neuromodulators such as ATP and NO [14]. These molecules can act upon neurons but also on glial cells reinforcing their reactivity. These reciprocal interactions between glial cells and neurons allow an amplification of the nociceptive information [15]. Therefore, thalamic-activated astrocytes could participate to the sensitization of VPL neurons to mechanical stimuli that has been recorded in peripheral neuropathic pain model [24, 26, 63].

### Temporal sequence of microglial and astrocytic alterations in the thalamus of SNL animals

It has been reported that activation of spinal microglia precedes the activation of spinal astrocytes in peripheral neuropathic pain model [7, 16, 64]. Furthermore, it has been recently shown that a subtype of reactive astrocytes can be induced by activated microglia [65], reinforcing the relevance of a microglial activation preceding an astrocytic activation. In the present study, we also found this typical temporal sequence of alteration at the thalamic level, even if the microglial alteration appears more complex and cannot unambiguously be assigned to a classical activation state. In the spinal cord, by using specific pharmacological inhibitors, spinal microglia have been implicated in the initiation phase of peripheral nerve injury-induced pain [18–21] while spinal astrocytes have been implicated in the maintenance of peripheral nerve injury-induced pain symptoms [10, 22]. It is tempting to propose that the same successive temporal role may be assigned to thalamic microglia and astrocytes in our SNL model. Of course, only pharmacological treatment impacting microglia or astrocytes function will shed light on the respective involvement of thalamic microglia and astrocytes on the onset and maintenance of neuropathic pain.

Our study raises the question of the mechanisms through which peripheral nerve injury could induce glial alteration in the thalamus which is far away from the site of injury. A previous study has provided evidence for remote microglial activation in the thalamus by the chemokine CCL21 after spinal cord injury and has demonstrated that CCL21 potently activates resident microglia within the VPL, which in turn pathologically modulate thalamic nociceptive neurons [66]. In this model, as the spinal cord is damaged, neurons of the spinothalamic tract that project to the thalamus could be damaged and therefore they could upregulate CCL21 and transport it to the thalamus. In the models of peripheral nerve injury (sciatic nerve transection, chronic constriction injury of the sciatic nerve, and spinal nerve ligation) in which thalamic glial alteration has been found [25, 27, 52, 53], it is necessary to postulate that injury to first-order nociceptive neurons can propagate signals to upstream targets, at least two synapses away in the thalamus. However what these signals are is a question that remains to be answered.

## Conclusion

Our results pave the way to decipher the role of astrocytes and microglia in the sensitization of the thalamic neurons to mechanical stimuli in this neuropathic pain model and may aid in the refinement of strategies that target glial activation to deal with neuropathic pain.

## Abbreviations

ANOVA: Analysis of variance
CCI: Chronic constriction injury
CCL21: Cysteine-cysteine chemokine ligand 21
CD11b: Cluster of differentiation molecule 11 b
CTSS: Cathepsin S
CX3CL1: C-X3-C motif ligand 1or fractalkine
CX3CR1: Fractalkine receptor
DAPI: 4′6′-diamidino-2-phenylindol
DWB: Dynamic weight bearing
GFAP: Glial acidic fibrillary protein
Iba-1: Ionized calcium binding adaptor molecule 1
IM: Intralaminar thalamic nuclei
MD: Mediodorsal thalamic nucleus
Q-PCR: Quantitative real-time PCR
SCI: Spinal cord injury
SNL: Spinal nerve ligation
TBS: Tris buffered saline
TLR4: Toll-like receptor 4
VPL: Ventral posterolateral thalamic nucleus
VPM: Ventral posteromedian thalamic nucleus
